# Malingering in the Emergency Setting

**DOI:** 10.7759/cureus.15670

**Published:** 2021-06-15

**Authors:** Tamar Zwick, Christopher Sharp, Daniel Severn, Scott A Simpson

**Affiliations:** 1 Psychiatric Emergency Services, Denver Health and Hospitals, Denver, USA; 2 Psychiatry, Atlanta Veterans Affairs Medical Center, Atlanta, USA; 3 Psychiatric Emergency Services, Denver Health Medical Center, Denver, USA

**Keywords:** malingering, emergency psychiatry, emergency treatment, hallucinations, suicide, suicidal ideations, emergency room, case study, feigned symptoms, homelessness

## Abstract

Malingering is the intentional fabrication of symptoms for material gain. Malingering among frequent utilizers and patients with psychiatric symptoms is suspected to be common in emergency settings but difficult to detect and manage. We present a case report of a 50-year-old man feigning psychosis and suicidality in order to obtain shelter. Strategies to identify malingered psychiatric symptoms are presented. Understanding how malingering is adaptational can help clinicians begin to manage these patients and symptoms in a compassionate manner that preserves healthcare resources, improves patient care, and reduces the risk of burnout for clinicians.

## Introduction

Malingering is the intentional fabrication or exaggeration of symptoms for material gain. Malingering is estimated to be present in up to 8% of all medical and psychiatric cases [1]. It is more common in certain settings such as criminal prosecutions and personal injury cases [2]. In emergency departments (EDs), the presence of malingering has been estimated to exceed 20%, particularly common among frequent utilizers and in psychiatric settings. True instances of malingering are likely higher than found diagnoses, given the challenges of diagnosis under the time constraints of emergency care [3-6]. Malingering has a significant financial impact in the United States where it is estimated to cost insurers approximately $150 billion dollars each year [7]. A review of Social Security Disability data suggests that malingering occurs in 46%-60% of adult mental disorder claimants at a cost of at least $20 billion per year [8]. Studies have found that 10%-12% of adults admitted for suicidality later endorse malingering [3]. In an era in which most municipalities report insufficient psychiatric beds, the use of scarce inpatient resources by malingering patients is particularly concerning. Because malingering is common in EDs, and many decisions regarding subsequent hospitalization and utilization are made in that setting, it is imperative that emergency clinicians recognize and manage malingered symptoms.

Here we present the case of a patient with malingered psychotic symptoms. We discuss the identification and management of malingered psychosis in the ED.

## Case presentation

A 50-year-old man presented to the emergency department (ED) complaining of auditory hallucinations (AHs) in the context of not taking his prescribed lurasidone and lorazepam. He initially denied suicidal and homicidal ideation to the triage nurse. While waiting for a room, he returned to the triage nurse saying he was suicidal and then brought back to a room for immediate evaluation. While in the medical ED, he began complaining additionally of a headache and homicidal ideation. The emergency medicine physician completed an unremarkable work-up including a head cranial tomography scan prior to a psychiatric assessment. The ED provider was highly concerned about the patient’s psychiatric presentation and advocated for psychiatric admission.

During the psychiatric evaluation, the patient was a vague historian, reporting “I don’t know how to do anything.” He often answered, “I don’t know,” and ascribed these responses to memory loss. He reported his hallucinations “tell me to do bad things, and I can’t control them.” The patient reported lorazepam no longer helped his symptoms, though also that he ran out. When asked where he stays at night, the patient answered, “emergency rooms.” Despite the patient’s report of severe AHs, he did not appear to be responding to internal stimuli or in observable distress. Over the course of the interview, the patient became increasingly hostile. He then demanded admission for treatment of alcohol withdrawal and stated that he was “DT-ing from alcohol,” despite a lack of clinical evidence of withdrawal symptoms. 

The psychiatric clinician reviewed the patient’s local medical records as well as those from other area hospitals. The patient’s record revealed near-daily ED visits; at an outside hospital, the week before the patient reported being prescribed escitalopram, alprazolam, and methylphenidate. A review of the prescription drug monitor program database revealed no outpatient prescriptions for these. His last inpatient discharge was one week prior; at that time treating providers observed that the patient often presented appropriately towards physicians but was disparaging and insulting towards ancillary staff. Prior clinicians diagnosed the patient with malingering and antisocial personality disorder.

On this visit, the psychiatry team strongly suspected malingering and found no emergent indication for treatment. The patient was discharged with a security escort given his hostility toward providers. The patient did not return to the ED for another month.

## Discussion

Malingering is a long-recognized phenomenon. Odysseus is said to have affected madness to avoid fighting in the Trojan War [9]. Today the Diagnostic and Statistical Manual of Mental Disorders (DSM-5) defines malingering as the “intentional production of false or grossly exaggerated physical or psychological symptoms, motivated by external incentives,” though its designation as a V code suggests it is not itself a diagnosis but rather an issue that may be a focus of clinical attention [10].

Detection of malingering is difficult, in part because of its episodic and situational nature [1]. Physicians are trained to detect illness rather than untruths. A 2006 meta-analysis found that despite additional training, “professional lie catchers” such as forensic psychologists and detectives were no more accurate at detecting deceptions than control evaluators [11]. Physicians are often concerned about the risk of liability, stigmatizing patients, or simply being wrong and missing a treatable illness; thus, clinicians are often reluctant to label behavior malingering even when suspected [12].

Conceptualizing malingering and the motivations behind it can be helpful for both detection and management. It is important to differentiate malingering from a similar diagnosis of factitious disorder. Intentional deception is an element of both diagnoses; however, the motivation differs. A person who is malingering is motivated by external incentives, while in the case of factitious disorder, "the deceptive behavior is evident even in the absence of obvious external rewards" [10]. Resnick proposed a model of malingering consisting of three subtypes: pure malingering - the complete fabrication of symptoms; partial malingering - the exaggeration of existing symptoms; and false imputation - the intentional attribution of symptoms to an unrelated cause [13]. The case above is consistent with a combination of partial malingering (the patient exaggerating the risk of alcohol or benzodiazepine withdrawal) and pure malingering, as was suspected of his subjectively reported suicidal ideation and hallucinations.

Malingering may be viewed as an adaptive mechanism that evolves as a response to significant stressors. This framework implies that patients are in need of services despite falsifying their presentations [7]. For example, one study examining an emergency setting found that almost half of those suspected of malingering had done so in order to obtain food or shelter, as in our case [5]. Other potential incentives include medications, financial gain, and avoidance of jail or other responsibilities. In this framework, malingerers engage in a “cost-benefit analysis” with individuals choosing to feign symptoms based on “their estimate of success in obtaining the desired external incentive”[14].

There have been no formal studies regarding the use of screening tools to detect malingering behaviors in the ED; however, there are two potentially useful questionnaires [15]. The Structured Interview of Reported Symptoms (SIRS) has been considered by many to be the gold standard in detecting psychiatric malingering since its publication in 1992. This structured interview consists of 172 verbally administered items and can typically be completed in 30-40 minutes; it has been reported to boast a sensitivity of 80% and specificity of 97% [16]. However, a 2011 meta-analysis suggests the specificity may be significantly lower than initially thought, estimating that the SIRS may fail to identify as many as one in four malingerers [16]. The Miller Forensic Assessment of Symptoms Test (M-FAST) may be more promising in emergency settings as it consists of only 25 items, can typically be administered in less than 15 minutes, and has fairly simple interpretation with yes or no responses and a cut-off value to indicate psychiatric malingering [15]. However, despite its potential, there is currently insufficient data supporting the clinical validity of the M-FAST outside of forensic settings [15, 17].

Even without formal testing, there are many signs which may indicate malingering. The DSM-5 states that malingering should be strongly suspected if any of the following are present: the medicolegal context of a presentation marked discrepancy between complaints and objective findings, lack of cooperation, and the presence of antisocial personality disorder [10]. Signs of malingering may include endorsement of symptoms that are rare, improbable, or contradictory to other elements of the history or mental status exam [17]. Indiscriminate symptom endorsement is also suspicious for malingering. Resnick suggests multiple techniques for identifying malingering, one of which is a detailed symptom inquiry [18]. Malingerers are unlikely to be as familiar with the subtle aspects of symptomatology and, following broad open-ended questions, should be probed for specific details, including rare or improbable symptoms. For instance, an evaluator might ask someone complaining of AHs if they see the words spelled out, which would be very unlikely. Improbable symptoms substantially raise the likelihood of malingering. A lengthy interview can also be an important component of the evaluation. Feigning symptoms often take significant effort; malingering patients are likely to grow tired as the interview progresses [14]. Asking repeated questions by multiple team members and by serial assessments over an extended period may reveal inconsistencies in a patient’s history. 

Collateral information is often critical in the detection of malingering by identifying secondary gain or revealing inconsistent details of the history, as in this case. A patient’s refusal to release such information should be documented as this behavior further supports malingering; in this case, substantial records were already available to the treatment team. This team benefitted from prior providers having thoughtfully evaluated the patient for malingering and carefully documented their observations in the medical record. Thorough documentation can both protect against litigation and aid clinicians with future evaluations [10].

One area in which malingering has received substantial investigation is psychosis, particularly establishing typical symptoms to distinguish true from malingered psychosis. For instance, a malingerer may report that AHs are unremitting, offer commands he is not capable of resisting, and sound as though they are coming from within his head such as in our case. However, most patients with true psychosis report that AHs are intermittent in nature, they have often developed coping skills to distract themselves from the voices, and more often than not they are able to resist commands [7,10]. The majority of patients with true psychosis report that AH sounds as though they are coming from outside of their heads [19]. A 2012 study found that only 34% of patients reported exclusively internal AH during their most recent episode with 66% reporting either external AH or both. Feigned AHs may also be more frightening, more unbearably distressing, and less predictable [20]. Visual hallucinations (VH) are almost always normal-sized people in regular colors [10]. A patient reporting visions of giant purple men is unlikely to be experiencing true psychosis. As with AHs, VHs are generally consistent with a patient’s delusional thought content. These fixed, false beliefs generally develop gradually and influence a patient’s behavior [7]. For example, a patient with paranoid delusions will typically be reluctant to offer details and guarded on the exam. Negative symptoms and thought disorganization are particularly difficult to feign and their absence in a patient complaining of hallucinations or delusions should raise suspicion of malingering. Table 1 describes more typical psychotic symptoms. When evaluating someone for malingered psychosis it is important to understand that the recognized phenomenology exists as a guide, rather than hard and fast rules; a single component in a thorough evaluation which, when combined with other evidence, can support malingering.

**Table 1:** Assessment of malingered psychosis.

	Symptoms	Typical	Atypical
Auditory hallucinations (AHs)			
	Frequency	Intermittent	Constant
	Associated with delusions	Yes	No
	Commands	Can resist AH	Must obey AH
	Coping strategies	Effective	Ineffective
	Negative	Yes	No
	Gender	Male and female	Single gender
	Identity	Able to identify	Unable to identify
Visual hallucinations (VHs)			
	Color	In color	Black and white
	Closing eyes	VH remain	VH cease
	Combination	AH +/- VH	VH alone
	Description	Lacks specificity	Very detailed
	Size of people	Normal	Abnormal (i.e., giants)
Delusions			
	Behavior	Consistent with delusions	Inconsistent
	Disorganization	Delusions + thought disorganization	Dramatic delusions with organized thoughts
	Details	Reluctant to share	Eager to share
	Onset	Gradual	Rapid
	Treatment response	Delayed	Rapid

Physicians may be uncertain of how to proceed clinically, even when malingering is strongly suspected. Clinicians may develop strong or varied countertransference to these patients; in this case, one clinician felt a strong personal connection to this patient. Such reactions are suspicious of predatory behavior by the patient. Lebourgeois suggests clinicians remember their “ABCS” when approaching patients who malinger - avoid (A) accusing the patient of lying, beware (B) of potential countertransference, seek clarification (C) rather than confrontation, and take necessary security (S) precautions beforehand [14]. It is important to clearly articulate realistic service options and to offer the patient “an out” by asking for any other potential causes, allowing the patient to “reveal their true need” [7]. As with many recommendations on managing malingering, these suggestions have yet to be empirically validated but are practically useful. Familiarity with identifying malingering and applying a framework to the care of these patients aids in reducing the risk of burnout and compassion fatigue for clinicians who see these patients frequently.

Finally, no discussion of malingering would be complete without considering the ethical implications. The principle of beneficence indicates that physicians have a duty to help their patients. Yet how does this apply to situations in which a patient is actively engaged in deception to obtain a resource that is not clinically warranted? Using the adaptation model, patients who malinger present with serious and genuine needs such as food and shelter. While rates of homelessness have decreased overall in the United States over the past decade, urban areas with higher housing costs have experienced significant increases in homelessness.

## Conclusions

Malingering of psychiatric symptoms is common in emergency settings. One can certainly understand - and indeed empathize with - an individual’s decision to feign symptoms in order to obtain shelter or food. Yet, that must be balanced with the probability that someone else will need the desired medical resource, including high-cost services such as hospitalization. Malingering is difficult to detect and thus requires substantial time, effort, and documentation by the clinician. However, doing so allows the clinician to recognize and focus on the patient’s real need in order to present them with realistic resources and appropriate treatment options.
